# Simple method of thawing cryo-stored samples preserves ultrastructural features in electron microscopy

**DOI:** 10.1007/s00418-020-01952-z

**Published:** 2021-01-06

**Authors:** Markus Galhuber, Nadja Kupper, Gottfried Dohr, Martin Gauster, Grazyna Kwapiszewska, Andrea Olschewski, Katharina Jandl, Elisabeth Gschwandtner, Martina Schweiger, Dagmar Kratky, Gerd Leitinger, Andreas Prokesch, Dagmar Kolb

**Affiliations:** 1 Gottfried Schatz Research Center for Cell Signaling, Metabolism and Aging, Division of Cell Biology, Histology and Embryology, Medical University of Graz, Neue Stiftingtalstraße 6/II, 8010 Graz, Austria; 2 Otto Loewi Research Center for Vascular Biology, Immunology and Inflammation, Division of Physiology, LBI for Lung Vascular Research, Medical University of Graz, Stiftingtalstrasse 24, 8010 Graz, Austria; 3 Division of Thoracic Surgery, Department of Surgery, Medical University of Vienna, Vienna, Austria; 4 Institute of Molecular Biosciences, Biochemistry II, University of Graz, Heinrichstraße 31/II, 8010 Graz, Austria; 5 Gottfried Schatz Research Center for Cell Signaling, Metabolism and Aging, Division of Molecular Biology and Biochemistry, Medical University of Graz, Neue Stiftingtalstraße 6/II, 8010 Graz, Austria; 6 BioTechMed-Graz, Mozartgasse 12/II, 8010 Graz, Austria; 7 Core Facility Ultrastructure Analysis, Neue Stiftingtalstraße 6/II, 8010 Graz, Austria

**Keywords:** TEM, Cryo-storage, Cryoprotectant-free, Ultrastructural features, Sample preparation, Biobanking

## Abstract

Preservation of ultrastructural features in biological samples for electron microscopy (EM) is a challenging task that is routinely accomplished through chemical fixation or high-pressure freezing coupled to automated freeze substitution (AFS) using specialized devices. However, samples from clinical (e.g. “biobanking” of bulk biopsies) and preclinical (e.g. whole mouse tissues) specimens are often not specifically prepared for ultrastructural analyses but simply immersed in liquid nitrogen before long-term cryo-storage. We demonstrate that ultrastructural features of such samples are insufficiently conserved using AFS and developed a simple, rapid, and effective method for thawing that does not require specific instrumentation. This procedure consists of dry ice-cooled pre-trimming of frozen tissue and aldehyde fixation for 3 h at 37 °C followed by standard embedding steps. Herein investigated tissues comprised human term placentae, clinical lung samples, as well as mouse tissues of different composition (brown adipose tissue, white adipose tissue, cardiac muscle, skeletal muscle, liver). For all these tissues, we compared electron micrographs prepared from cryo-stored material with our method to images derived from directly prepared fresh tissues with standard chemical fixation. Our protocol yielded highly conserved ultrastructural features and tissue-specific details, largely matching the quality of fresh tissue samples. Furthermore, morphometric analysis of lipid droplets and mitochondria in livers of fasted mice demonstrated that statistically valid quantifications can be derived from samples prepared with our method. Overall, we provide a simple and effective protocol for accurate ultrastructural and morphometric analyses of cryo-stored bulk tissue samples.

**Supplementary Information:**

The online version contains supplementary material available at 10.1007/s00418-020-01952-z.

## Introduction

**Fig. 1:**
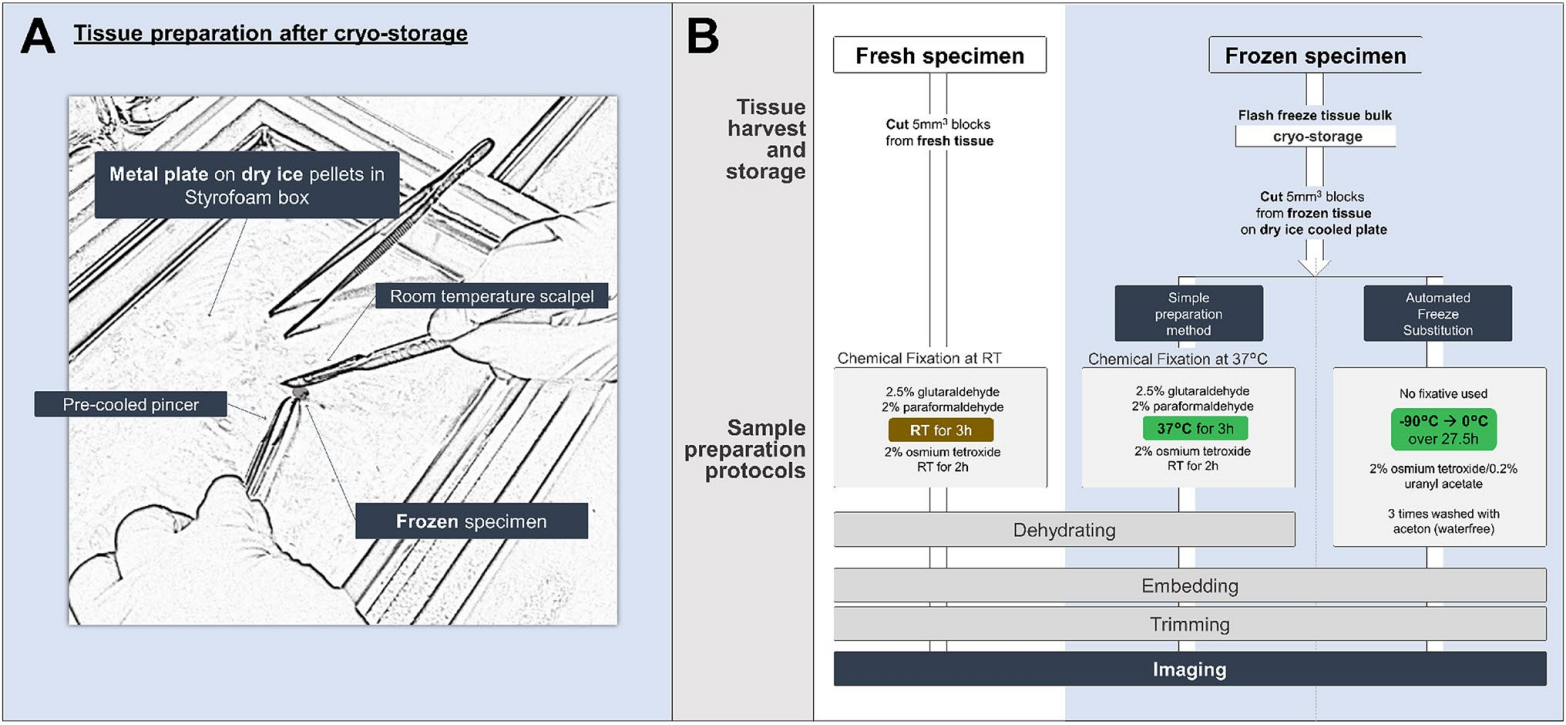
Sample preparation and processing. **a** The preparation of ~ 5mm^3^ tissue specimens from frozen bulk tissues for ultrastructural imaging was conducted on a pre-cooled metal plate placed on a layer of dry ice pellets with pre-cooled forceps and a room temperature scalpel. **b** Fresh tissue samples/biopsies were cut into ~ 5mm^3^ blocks and immediately after dissection, chemically fixated at room temperature, dehydrated, embedded in TAAB resin and trimmed for ultrastructural imaging. Tissues/biopsies for cryo-storage were dissected and the bulk tissue immediately flash frozen in liquid nitrogen without cryo-preserving agents. Frozen tissue samples were cut into ~ 5mm^3^ pieces and either fixated at 37 °C in 2.5% (wt/vol) glutaraldehyde and 2% (wt/vol) paraformaldehyde or underwent a standard protocol for automated freeze substitution. Downstream processing for imaging was conducted similar to the fresh tissue control group

From its inception in the 1930’s (Ruska 1987), transmission electron microscopy (TEM) has facilitated life sciences early on with crucial insights such as the prove for the existence of viruses (Kausche et al. 1939) and the first images of cellular substructures (Harris 2015). Today, specialized applications like cryo-TEM (or cryo-EM) aid the determination of macromolecular structures at near-atomic resolution (Nogales and Scheres 2015). Besides steady improvements on hardware and software, advances in sample preparation techniques that conserve in situ structures have propelled the field forward. Detailed analyses and improvements of all steps in the preparation procedure have been instrumental to reduce artifacts that limit resolution and contrast of electron micrographs (Mollenhauer 1993). Conventional preparation of fresh biological specimens for TEM analyses is done by chemical fixation with aldehydes and osmium tetroxide, followed by dehydration, embedding, and trimming. As an alternative to chemical fixation, methods of cryofixation were devised (Plattner and Bachmann 1982) that have the advantage of a much higher fixation rate. However, application of cryofixation is severely limited to a sample thickness of about 10–40 µm, below which abundant ice-crystal formation leads to precipitation of macromolecules as well as phase segregation (Plattner and Bachmann 1982). To minimize such freezing artifacts, cryoprotectants (e.g. dimethyl sulfoxide (DMSO)) can be used to vitrify hydrated samples (Fahy and Wowk 2015), although these substances introduce changes to cell and tissue morphology on their own (Dahl and Staehelin 1989). High-pressure freezing (HPF) with subsequent automated freeze substitution (AFS) markedly improves the artifact-free surface layer depth to 0.2 mm without the need of cryoprotectants (Dahl and Staehelin 1989; Studer et al. 2008b). However, HPF demands highly specialized devices that can apply liquid nitrogen at a pressure of 210 MPa. For most clinical samples in biobanks (Coppola et al. 2019) or tissues from animal experiments that are stored in agreement with the 3R’s of animal experimentation (Sneddon et al. 2017), the above-mentioned biophysical considerations have not been taken into account upon freezing and procedures to avoid a range of ultrastructural artifacts are not applicable. Hence, in our electron microscopy facility, we are often confronted with clients that ask for retrospective ultrastructural analyses of bulk tissue parts and biopsies that were frozen through submersion in liquid nitrogen and then cryo-stored (− 196 °C for liquid nitrogen or − 80 °C in laboratory freezers) without initially intending downstream TEM analyses. To retrieve accurate ultrastructural information from such cryo-stored bulk tissues, we aimed for a thawing method that (1) works for samples simply immersed in liquid nitrogen before storage, (2) does not demand pre-treatment (e.g. addition of cryo-protectants), (3) minimizes preparation time, (4) is not restricted by shape and composition of the sample, and (5) is independent of expensive, highly specialized instrumentation. Here, we compare electron micrographs of cryo-stored samples processed with our simple preparation method by subjecting them to chemical fixation at 37 °C upon thawing or prepared from fresh tissue samples through chemical fixation at room temperature (Fig. 1). Although our presented method provides high-quality electron micrographs from frozen bulk tissue, we want to emphasize that the optimal strategy for clinical diagnostics on the ultrastructural level is still HPF with subsequent freeze substitution (Dahl and Staehelin 1989; Studer et al. 2008a).

## Materials and methods

### Cryo-preservation procedure

Tissue samples (mouse: brown adipose tissue, epididymal white adipose tissue, cardiac muscle, skeletal muscle, liver; human: placenta, lung) were cut into approximately 5mm^3^ sized pieces, briefly washed in PBS pH7.4, transferred into a dry 1.5 ml Eppendorf Safe-Lock tubes and immediately immersed into liquid nitrogen. Samples were either stored in liquid nitrogen or at minus 80 °C.

### Harvesting of mouse tissues

Mice were housed under standard 12-h light/12-h dark cycles. For feeding studies, wild-type C57Bl/6 N mice were housed individually in grid-bottom cages and either fed ad libitum (standard chow) or fasted for 24 h prior to study. For both groups water was available ad libitum. After euthanizing the animals, intact organs or tissues (liver, epididymal white adipose tissue, interscapular brown adipose tissue, skeletal muscle, cardiac muscle) were immediately dissected, briefly washed in PBS pH 7.4, transferred into a dry 1.5 ml Eppendorf Safe-Lock tube, immediately immersed in liquid nitrogen, and stored without addition of cryo-preservant at minus 80 °C.

### Specimen preparation of frozen/liquid nitrogen immersed samples

Intact organs/tissues/biopsies were taken from either minus 80 °C freezer or liquid nitrogen storage and placed on a dry-ice-cooled metal plate in a Styrofoam box filled with dry ice (Fig. 1a). Tissue was pinned down with pre-cooled forceps and sectioned with a room temperature scalpel to produce tissue blocks of about 5 mm^3^. The use of a room temperature scalpel is necessary to prevent splintering and adhesion of the sample pieces to the scalpel. The obtained frozen tissue sections were submerged in fixative (2.5% (wt/vol) glutaraldehyde and 2% (wt/vol) formaldehyde, buffered in 0.1 M cacodylate buffer, pH 7.4 and incubated at 37 °C for 3 h (Fig. 1b). Samples can be optionally transferred into 0.1 M cacodylate buffer and incubated overnight. Post-fixation in 2% osmium tetroxide (diluted in 0.2 M cacodylate buffer) for 2–3 h at room temperature. After washing for 2 h in 0.1 M cacodylate buffer, the specimens were dehydrated in a graded series of ethanol (50%, 70%, 80%, 96%, 100% p.a.), infiltrated with propylene oxide/TAAB (Agar Scientific, Essex, GB) embedding resin (propylene oxide 1 h room temperature, propylene oxide/TAAB 1:1 3 h room temperature, propylene oxide/TAAB 1:3 o/n 4 °C) and finally embedded in pure resin and polymerized (2 × 1.5 h 48 °C).

### Conventional specimen preparation of fresh samples

Freshly harvested tissues were immediately fixed in 2.5% (wt/vol) glutaraldehyde and 2% (wt/vol) paraformaldehyde, buffered in 0.1 M cacodylate buffer, pH 7.4 and incubated at room temperature for 3 h. Samples can be optionally transferred into 0.1 M cacodylate buffer and incubated overnight. Post-fixation in 2% osmium tetroxide (diluted in 0.2 M cacodylate buffer) for 2–3 h at room temperature. Workflow see Fig. 1b. After washing for 2 h in 0.1 M cacodylate buffer the specimens were dehydrated in a graded series of ethanol (50%, 70%, 80%, 96%, 100% p.a.), infiltrated with propylene oxide/TAAB (Agar Scientific, Essex, GB) embedding resin (propylene oxide 1 h room temperature, propylene oxide/TAAB 1:1 3 h room temperature, propylene oxide/TAAB 1:3 o/n 4 °C) and finally embedded in pure TAAB resin and polymerized (2 × 1.5 h 48 °C).

### Automated freeze substitution (AFS) of frozen/liquid nitrogen immersed samples

Frozen samples were pre-trimmed similar to the specimen preparation on a dry-ice-cooled metal plate and the obtained frozen tissue pieces prepared as previously described (Sele et al. 2019). In brief, samples were incubated for 8 h at − 90 °C in 2% osmium tetroxide/0.2% uranyl acetate (diluted in water free acetone) within an AFS2 (Leica Microsystems, Vienna, Austria) (Fig. 1b). The samples were then gradually warmed up to − 60 °C with an increase of 30 °C per hour and maintained at this temperature for 8 h. Afterwards, the temperature of the samples was gradually raised to − 30 °C with an increase of 30 °C per hour and maintained for 8 h. In a last step, samples were warmed from − 30 °C to 0 °C over a period of 1.5 h. Finally, samples were washed three times in water free acetone at room temperature and then placed into aceton/TAAB 1:1 for 3 h room temperature, followed by aceton/TAAB 1:2 o/n 4 °C. After two pure TAAB steps for 1.5 h at 45 °C each the samples were finally embedded in pure TAAB and polymerized at 60 °C for 3 days.

### Ultrathin sectioning

Semi-thin and ultrathin sectioning was conducted as previously described (Sele et al. 2019). In brief, semi-thin sections were acquired and stained with a 1% toluidine blue solution (Sigma-Aldrich, USA) to confirm the area of interest under the light microscope (Olympus BX63) for trimming. Ultra-thin sections were cut on an ultramicrotome UC7 (Leica, Vienna, Austria) at 70 nm and collected on copper grids.

### Electron microscope image acquisition

Ultrathin sections were stained with platinum blue (Inaga et al. 2007) for 15 min and lead citrate for 7 min. Imaging was performed as previously described (Kolb et al. 2014). Images were taken at 120 kV with a Tecnai G2 FEI (Thermo Fisher Scientific) microscope equipped with an Ultrascan 1000 CCD camera (Gatan).

### Morphometric analyses of lipid droplets and mitochondria

Several electron micrographs (2500 × magnification) were obtained from liver pieces of ad libitum fed (*n* = 3) and starved (*n* = 3) mice in a randomized fashion, amounting to a total of 95 (fed) and 100 (starved) images of hepatocytes from each group. Images were analyzed with the “Image Analysis” module of the imaging software Zeiss Zen (blue edition) to count the number of morphologically intact mitochondria and lipid droplets, while disregarding truncated mitochondria and lipid droplets at the boarders of the micrographs. Morphologically intact mitochondria were defined as showing a clearly visible double membrane and observable cristae. In a second step, ImageJ software (Image Processing and Analysis in Java) (Schindelin et al. 2015) was used to quantify the diameter and number of lipid droplets. The resulting data were collected in Microsoft Excel to compile statistics of the quantity of intact mitochondria and lipid droplets. Figures were produced with the GraphPad Prism 8 software.

## Results and discussion

**Fig. 2:**
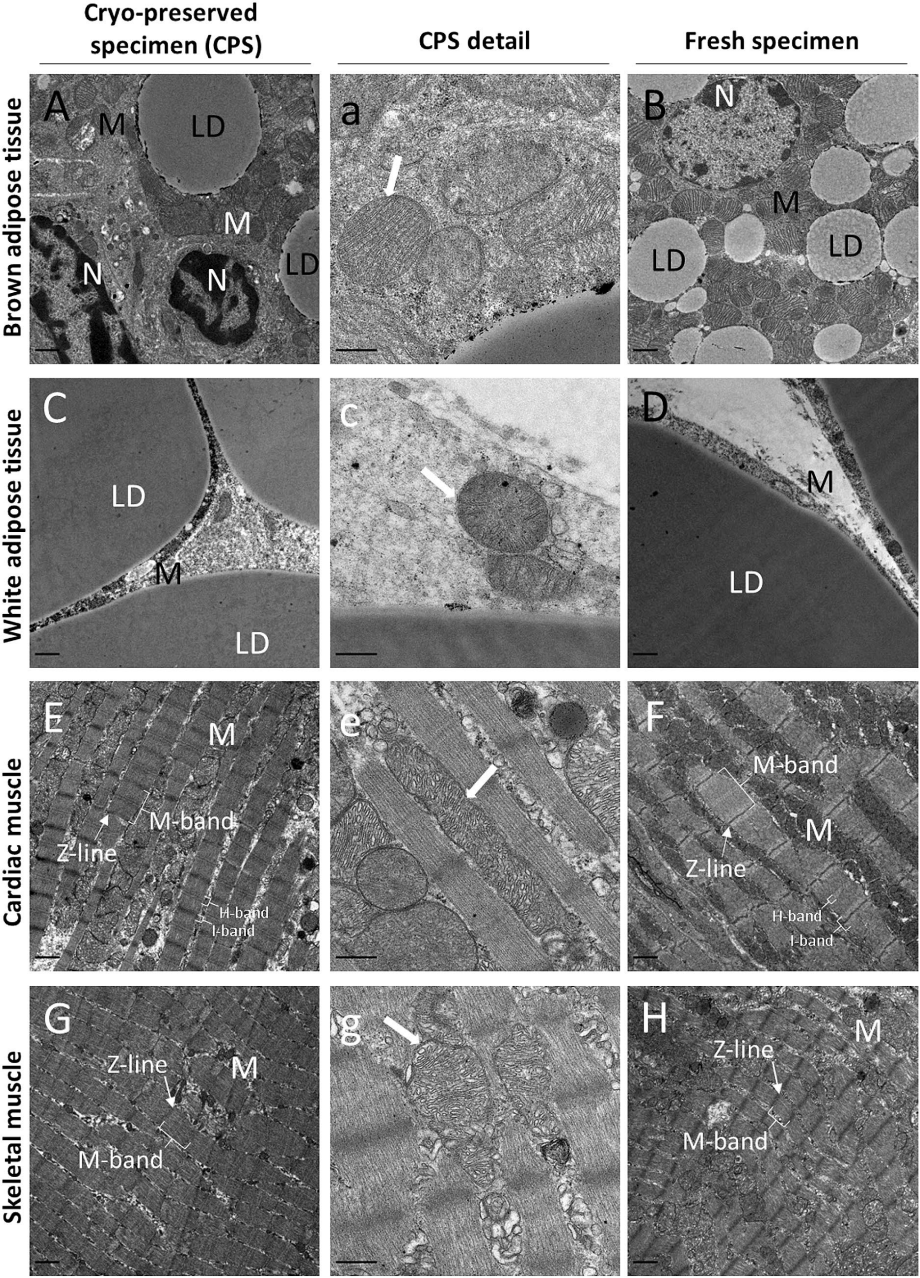
Electron micrographs of cells from cryo-preserved and conventionally prepared mouse tissues show comparable histomorphological features. **A**, **a**, **B** BAT cells show typical ultrastructural features, i.e. large, round-shaped mitochondria with packed cristae and small multilocular lipid droplets. **C**, **c**, **D** Epididymal WAT characterized through one unilocular large lipid droplet. Nucleus and cytoplasm characteristically forced to peripheral areas. **E**, **e**, **F** Longitudinal section of cardiac muscle fibers organized in branched sarcomers, each defined by perpendicular Z-lines, and interspaced with numerous mitochondria. Filament-associated features, i.e. M-band, I-band and H-band are clearly visible. **G**, **g**, **H** Parallel myofibrils in longitudinal section in skeletal muscle encased with sarcoplasmic reticulum. Higher magnified micrographs **a**, **c**, **e**, **g** show double membranes (bold white arrows) of mitochondria for each tissue. LD…lipid droplets M…mitochondria N…nucleus. Scale bars indicate 1 µm

**Fig. 3:**
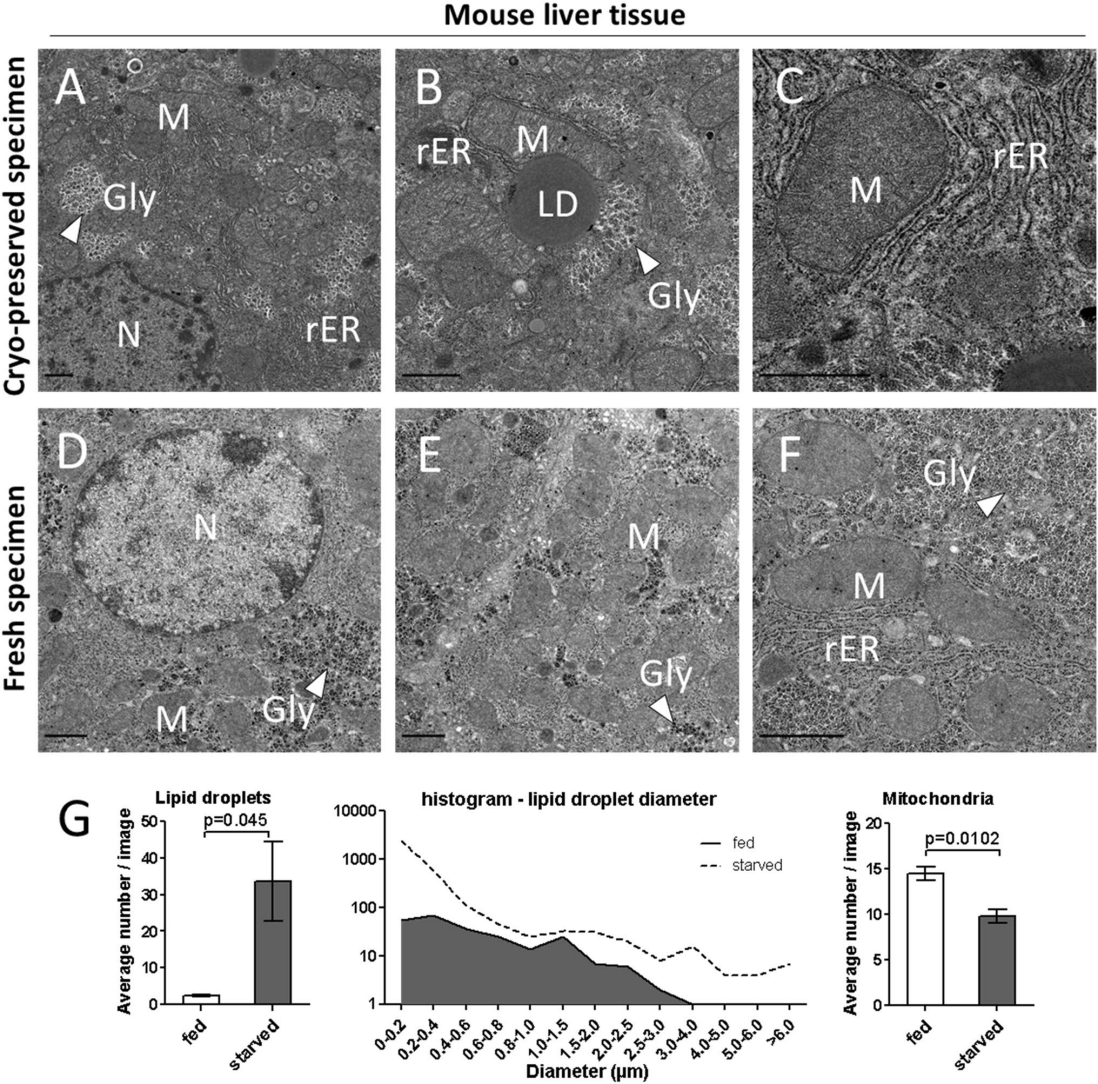
Electron micrographs of mouse liver of 24 h fasted mice in different magnifications. **a**, **b**, **c** EMs of cryo-preserved samples. **a** Hepatocyte shows a euchromatic nucleus (N), stacked rough endoplasmic reticulum (rER), round and elongated mitochondria (M), glycogen areas (indicated with white arrow head). **b** Large lipid droplet (LD) attached to mitochondrion with partially conserved shelf-like cristae. Many individual glycogen particles cluster together forming rosettes (white arrowheads) in the cytoplasm. **c** Mitochondria in vicinity of stacked rough endoplasmic reticulum. **d**, **e**, **f** EMs of freshly prepared samples. **d** Hepatocyte shows a euchromatic nucleus (N). Mitochondria (M) and glycogen clusters (white arrowheads) are indicated. **g** (Left panel) The average number of lipid droplets per micrograph significantly increased in starved animals over fed animals (*p* = 0.045). (Middle panel) Lipid droplet distribution is blotted as lipid droplet diameter (horizontal axis) against its frequency. Starved animals (dashed line) show a higher overall abundance of lipid droplets as well as markedly increased of small diameter droplets. Concomitantly, the average number of mitochondria per image was significantly reduced in mouse liver micrographs from starved animals (24 h food withdrawal) in comparison to fed animals (*p* = 0.0102) (Right panel). Scale bars indicate 1 µm

**Fig. 4:**
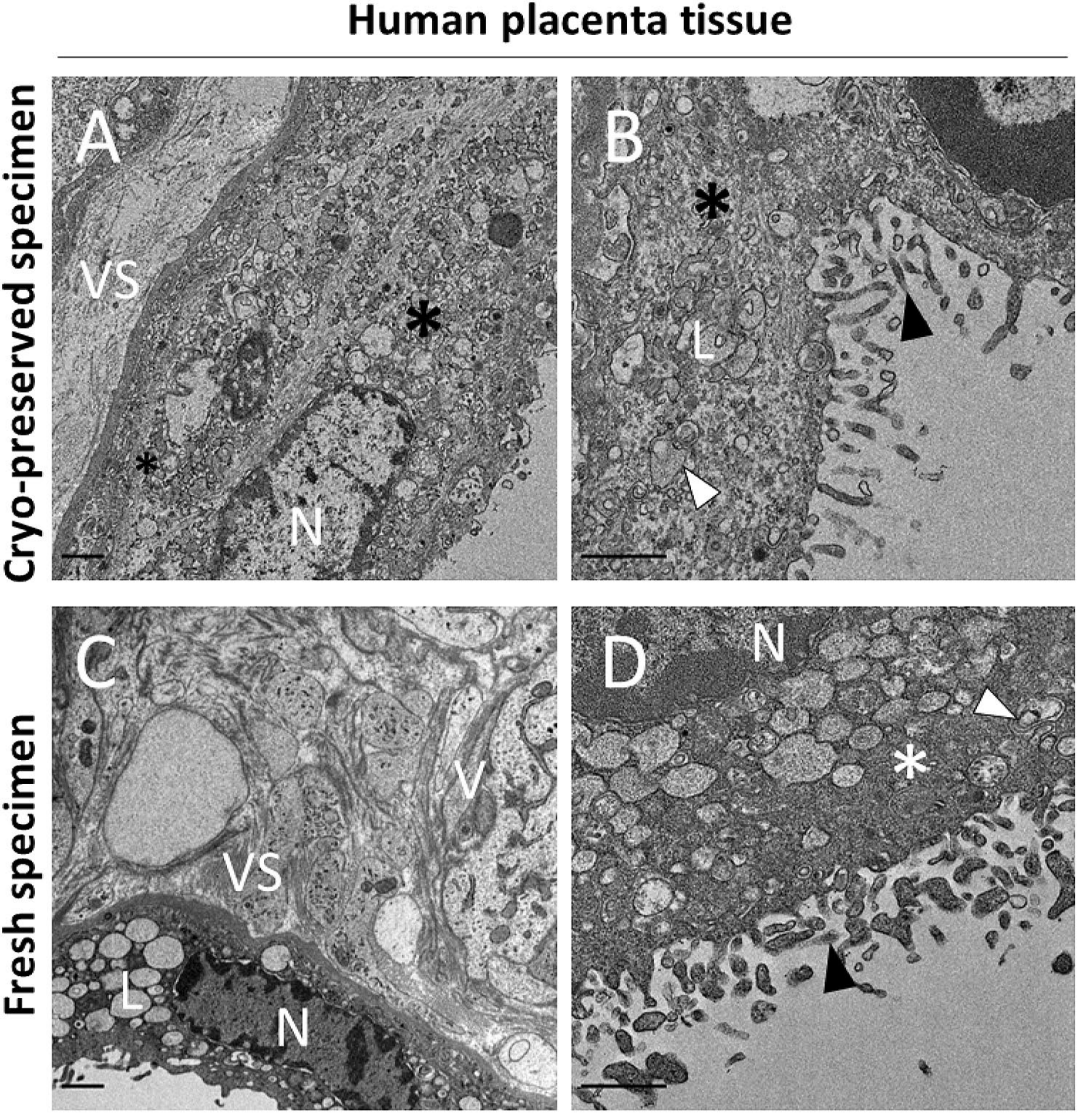
Electron micrographs of human placenta tissue from one placenta sample in different magnifications. **a**, **b** EMs of cryo-preserved samples. **a** Cryofixed placenta tissue with syncytiotrophoblast layer (black asterisks), nucleus (N), villous stroma (VS). **b** Microvilli (black arrowhead) at the apical part of syncytiotrophoblast, lysosomes and late autophagic compartments (white arrowheads) are well preserved regardless of preparation method (**b** and **d**). **c**, **d** EMs of freshly prepared samples. **c** EM shows villous stroma (VS) with collagen fibres, lysosomes (L) and nucleus (N) with heterochromatin, **d** Endosomes and late autophagic compartments (white arrowhead) within the syncytiotrophoblast (white asterisk); microvilli (black arrowhead) located at the apical side. Scale bars indicate 1 µm

**Fig. 5:**
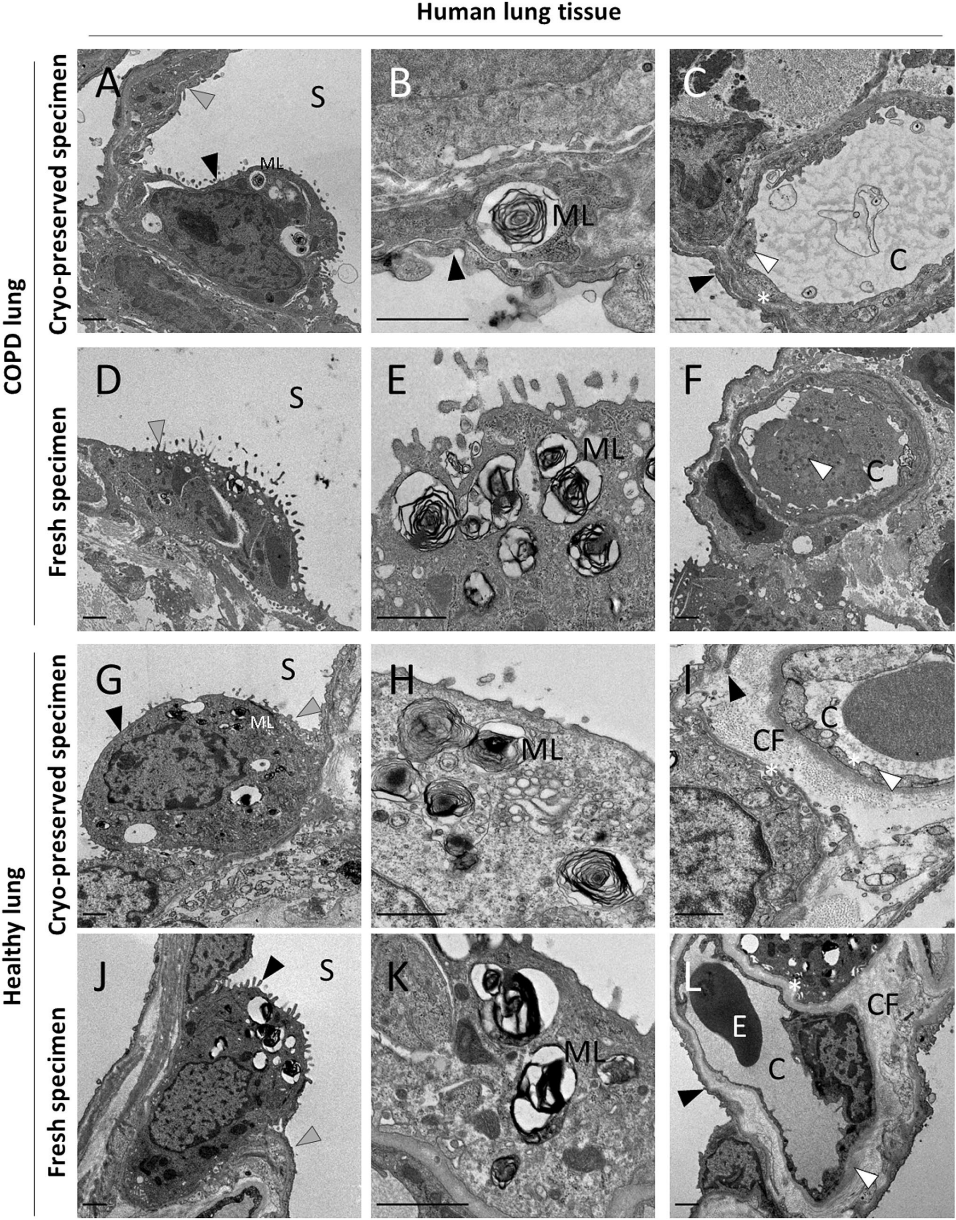
Electron micrographs of COPD and non-COPD/non-cancerous lung tissue in different magnifications. **a**, **b**, **c** EMs of cryo-preserved COPD samples show similar quality to freshly prepared samples. **a** Micrograph showing a Type II pneumocyte (cuboidal cell, black arrowhead) and the large Type I pneumocyte (grey arrowhead) lining the alveolar space (S); multilamellar bodies (ML) are visible within the Type II pneumocyte. **b** Higher magnification of multilamellar body (ML) with dense membrane. **c** Capillary (C) shows well-preserved endothelium (white arrowhead), epithelium (black arrowhead) and basement membrane (asterisk) (**d**–**f**) Micrographs of freshly prepared COPD samples. **d** Type I Pneumocyte (grey arrowhead). **e** Higher magnification of multilamellar bodies (ML) within Type II pneumocyte. **f** Capillary (C) with two blood cells (white arrowhead) in close proximity to the alveolar space. (**g**–**i**) EMs of cryo-preserved non-COPD/non-cancerous samples show similar quality to freshly prepared samples. **g** Micrograph showing a Type II pneumocyte (cuboidal cell, black arrowhead) and the large Type I pneumocyte (grey arrowhead) lining the alveolar space (S); multilamellar bodies (ML) are visible within the Type II pneumocyte. **hh** Higher magnification of multilamellar bodies (ML) with dense membrane stacks. **i** Capillary (C) shows well-preserved endothelium (white arrowhead), epithelium (black arrowhead) and basement membrane (asterisk) Collagen fibers (CF) are well preserved. (**j**–**l**) Micrographs of freshly prepared non-COPD/non-cancerous samples. **j** Type I Pneumocyte (grey arrowhead) and Pneumocyte II (black arrowhead) lining the alveolar space (S). **k** Higher magnification of multilamellar bodies (ML). **l** Capillary (C) with Erythrocyte (E), endothelium (white arrowhead) and epithelium (black arrowhead) are shown. Basement membrane (white asterisk) and collagen fibers (CF) are well preserved. Scale bar length 1 µm

In this study, tissue samples of human and murine origin were processed for TEM. The tissues were either fixed and embedded immediately after harvesting from the source (“fresh”) or prepared from cryo-stored bulk tissue immersed in liquid nitrogen (“frozen”). For frozen tissue samples we applied our preparation method as well as AFS without preceding HPF. We scrutinized whether electron micrographs from frozen samples prepared with our method would yield comparable histo-morphological quality when compared to micrographs of freshly prepared tissue sections (Figs. 2, 3, 4, 5) or frozen samples processed with AFS (Supplemental Figures S1 – S3). In contrast to conventional fixation or another published method fixating at 4 °C (Fortunato et al. 2016), our preparation method for cryo-stored samples uses a higher temperature (37 °C instead of room temperature as normally used in non-frozen samples) during chemical fixation, with the rationale to facilitate an accelerated thawing process. Both, frozen as well as fresh samples which underwent chemical fixation were fixated in 2.5% glutaraldehyde/2% paraformaldehyde (Fig. 1). Frozen samples processed with AFS were not chemically fixated. Toluidine blue staining and electron micrographs processed with AFS showed high levels of degradation and overall inferior tissue quality (Supplement Figs. S1–S3). The highly degraded appearance of the analyzed tissues suggests that AFS (Mcdonald and Webb 2011) is not suitable for bulk tissue samples which were not previously prepared with specialized methods like high-pressure freezing (Studer et al. 2008b).

### Ultrastructure of mouse tissues upon different sample preparation methods

As AFS is not amenable for flash frozen bulk tissue (shown in Supplement Figs. S1–S3), we compared electron micrographs generated from samples prepared with our method for cryo-stored tissues with electron micrographs from fresh, chemically fixated tissues. We first evaluated images from murine brown adipose tissue (BAT), epididymal white adipose tissue (eWAT), cardiac muscle, and skeletal muscle (Fig. 2; for comparison AFS-prepared electron micrographs from mouse tissues are given in Supplemental Fig. 1). The ultrastructure of all these tissues prepared with our method showed highly comparable quality between images from fresh and stored samples. Characteristic ultrastructural features of brown and white adipocytes, especially lipid droplets and mitochondria, were well conserved in both cases and did not show any sign of ice-crystal formation. Higher magnifications show that electron micrographs derived with our method allows detailed study of structures as small as mitochondrial cristae (Fig. 2, middle panel). BAT showed typical features like a central nucleus, abundant mitochondria with packed cristae, and multilocular lipid droplets (Cigolini et al. 1986; Zancanaro et al. 1995). With both preparation methods, eWAT showed well-preserved large lipid droplets surrounded by a corona of cytoplasm including small mitochondria. For BAT and eWAT, we observe slight variation in electron densities of lipid droplets, which could be attributable to our preparation method. Cardiac and skeletal muscle show clear sarcomer structures with distinct line and band compartmentation, indicative of synchronized actin and myosin arrangements. Substructures such as Z-lines within A-bands and M-bands within I-bands are clearly visible in both preparations. Furthermore, mitochondria are abundant and well-preserved between sarcomeres in cardiac muscle tissue. We conclude that preparation of cryo-stored, frozen specimens with our method of thawing at 37 °C in fixative derives electron micrographs of comparable quality to electron micrographs from fresh specimens.

To further confirm the utility of our method for frozen tissue, we next investigated mouse liver tissue and compared electron micrographs from frozen liver specimens to freshly prepared samples. Complementary AFS-prepared EMs from mouse livers are shown in Supplemental Fig. 2 and were assessed as not suitable for our intended downstream analysis. Figure 3 shows three different magnifications of mouse liver tissue from ad libitum fed mice. Frozen specimens (Fig. 3a–c) show typical features of hepatocytes like euchromatic nuclei, rough endoplasmic reticulum, as well as rounded and elongated mitochondria. Lipid droplets are scattered within the cytoplasm and partially attached to mitochondria (Fig. 3b). Patches of clustered glycogen particles are preserved and visible as rosettes in the cytoplasm. In comparison to specimen prepared from fresh samples (Fig. 3d–f), we do not observe impaired quality of the frozen specimens, strongly arguing for comparability of our preparation methods.

### Quantification of mitochondria/lipid droplets in liver

Based on the ultrastructural comparability of fresh and frozen samples processed with our simple method, we were confident to use frozen mouse liver tissues and analyze lipid droplet and mitochondria distribution under different nutritional conditions (ad libitum chow diet vs. fasting for 24 h). We hypothesized that we can reproduce previously described ultrastructural adaptation to nutrient depletion in mouse liver tissue under fasting (Guan et al. 2009) by investigating lipid droplet number and mitochondrial frequency in mouse hepatocytes. Furthermore, lipid droplet size distribution was analyzed and compared between ad libitum fed and fasted mice.

In total, 95 micrographs with a magnification of 2500 × were recorded for the ad libitum fed mice, and 100 micrographs were obtained for the 24 h starved cohort resulting in a total analyzed area of 6126 or 6448 µm^2^. In both groups, three different tissue blocks from three different mice were investigated. Randomly recorded, high-quality micrographs were then analyzed and counted manually for lipid droplets and mitochondria.

The fasting regime promoted a significantly (*p* = 0.045) increased number of lipid droplets per analyzed image (33,7lds/image) compared to fed control group (2,5lds/image) (Fig. 3g left panel). Assessing the size distribution of the lipid droplets, we observed that not only the total number of lipid droplets increase upon starvation, but also smaller sized lipid droplets highly increase in number. Based on that observation, we systematically evaluated LD sizes and blotted the size distribution for both experimental conditions in a histogram (Fig. 3g middle panel). We found that the area totally covered by lipid droplets significantly increased under fasting conditions in comparison to the ad libitum fed group. This accumulation of lipid droplets in hepatocytes after 24 h of fasting has been shown before, and is believed to be an intracellular mechanism to protect from lipotoxicity (i.e. high intracellular FFA levels) when systemic FFA levels are massively induced in fasted C57Bl/6 mice (Guan et al. 2009).

Furthermore, mitochondrial abundance and morphology is tightly controlled under stress conditions. Early adaptation to starvation conditions invokes mitochondrial (hyper) fusion, which is ultrastructurally reflected in an elongated shape, sometimes even resulting in reticular networks of mitochondria. This mitochondrial remodeling is interpreted as protective morphological shape change to prevent unwanted elimination through non-selective autophagy (Tondera et al. 2009; Rambold et al. 2011; Wai and Langer 2016) and might lead to an observable reduction in their abundance in ultrastructural micrographs. To analyze this, we systematically evaluated the number of mitochondria in our micrographs. The fasting regime promoted a significantly (*p* = 0.0102) reduced number of observable mitochondria per analyzed image (average 9,8 mt/image) compared to fed control group (average 14.5 mt/image) (Fig. 3g right panel). This reduction reflects previously published results of reduced mitochondrial abundance under prolonged starvation conditions in mouse cells (Rambold et al. 2011). Hence, we were able to reproduce morphological/physiological correlates, observed in micrographs from fresh specimen, also in frozen mouse liver sections. Together, these data underline that our method is suitable for morphometric analyses of tissues immersed in liquid nitrogen with TEM.

### Ultrastructure of human placenta and lung tissues upon different sample preparation methods

To extend our analysis to human specimens, we compared the quality of micrographs from fresh versus bio-banked placental and lung tissue. Furthermore, we compared our sample preparation method to AFS-prepared samples from the identical placental and lung tissues (AFS EMs see Supplemental Figure S3) and concluded that AFS for liquid nitrogen-submerged tissue samples was not suitable for ultrastructural analysis.

To assess ultrastructural quality of human placenta tissue, a sample from the same donor was either fixed immediately or immersed in liquid nitrogen and analyzed after preparation according to Fig. 1. Our obtained micrographs from frozen and fresh placenta tissue (Fig. 4a–d) show accurately preserved autophagic compartments underneath the microvillous plasma membrane of the syncytiotrophoblast. Also, microanatomical features such as microvilli, lysosomes, heterochromatic parts in the nucleus, and high number of vesicles are clearly visible. Based on our analysis, we do not observe any substantial alterations in the structural organization of the placental microstructure in cryopreserved samples in comparison to freshly prepared tissue samples. We, therefore, conclude that retrospective analysis of bio-banked pathological cases such as preeclampsia and interuterine growth restriction (IUGR) would be feasible with our method.

We further asked if cryo-stored biobank samples from chronic obstructive pulmonary disease (COPD) patients could be used for ultrastructural analysis and compared them to fresh specimen preparation and to samples from a healthy lung tissue prepared with both methods. COPD is a pulmonary disease that poses a severe public health problem with a high morbidity and mortality (Tuder and Petrache 2012; Khakban et al. 2017). Given its high prevalence, deciphering the underlying pathological processes is of great importance, but availability of fresh lung tissue is often limited. Preparation of lung tissue for ultrastructural analysis is generally a challenging task, as even in healthy conditions, its spongy, air-filled consistency renders it prone to preparation artifacts. In the case of COPD, injury events, increased apoptosis, and matrix remodeling lead to alveolar destruction, further complicating tissue preservation (in our observations). Despite these challenges, our results demonstrate that the ultrastructure of both, fresh and frozen COPD as wells as healthy donor lung samples show characteristic features and are of comparable quality (Fig. 5). We observed a preserved structure on multiple levels: (1) on a cellular basis as demonstrated by flat, small nuclear type 1 pneumocytes lining the alveolar wall; (2) on an intracellular level, by preservation of organelles, such as lamellar bodies in type 2 pneumocytes; and (3) on extracellular level by displaying an intact fused basement membrane connecting the endothelium and the epithelium.

## Conclusion

Based on our comparative study, we conclude that frozen tissue samples from biobanks can be used to assess ultrastructural morphology when processed with our method, which drastically extends the spectrum of samples for retrospective ultrastructural analyses. Overall, simple thawing of frozen human tissue at 37 °C in fixative renders tissues frozen in liquid nitrogen immediately after collection (e.g. placental tissue after delivery) amenable for ultrastructural analysis with TEM. Finally, we want to emphasize that the optimal strategy for clinical diagnostics on the ultrastructural level is still HPF with subsequent freeze-substitution, while our method seems ideal for bio-banked bulk tissue samples.

## Supplementary Information

Below is the link to the electronic supplementary material.

Supplementary Figure S1 Toluidine blue staining and electron micrographs from cryo-preserved mouse samples after preparation with AFS. (A, B) brown adipose tissue. (C, D) white adipose tissue. (E, F) cardiac muscle. (G, H) skeletal muscle. Microscopic pictures show partly intact tissue structure with Toluidine blue staining (20X magnification), while electron micrographs show highly degenerated ultrastructure. Scale bar length 1µm (TIF 11955 KB)

Supplementary Figure S2 Electron micrographs from cryo-preserved mouse liver samples after preparation with AFS. (A, B) mouse liver fed. (C, D) mouse liver fasted. Toluidine blue staining 20X magnification. Scale bar length 1µm (TIF 7053 KB)

Supplementary Figure S3 Electron micrographs from cryo-preserved human samples after preparation with AFS. (A, B) human placenta tissue. (C, D) human lung tissue. Toluidine blue staining 20X magnification. Scale bar length 1µm (TIF 5329 KB)
