# Supplementary figures and images for: Simple method of thawing cryo-stored samples preserves ultrastructural features in electron microscopy
Source: Histochem Cell Biol. 2021 Jan 6;155(5):593–603. doi: 10.1007/s00418-020-01952-z (PMC8134286; doi:10.1007/s00418-020-01952-z)

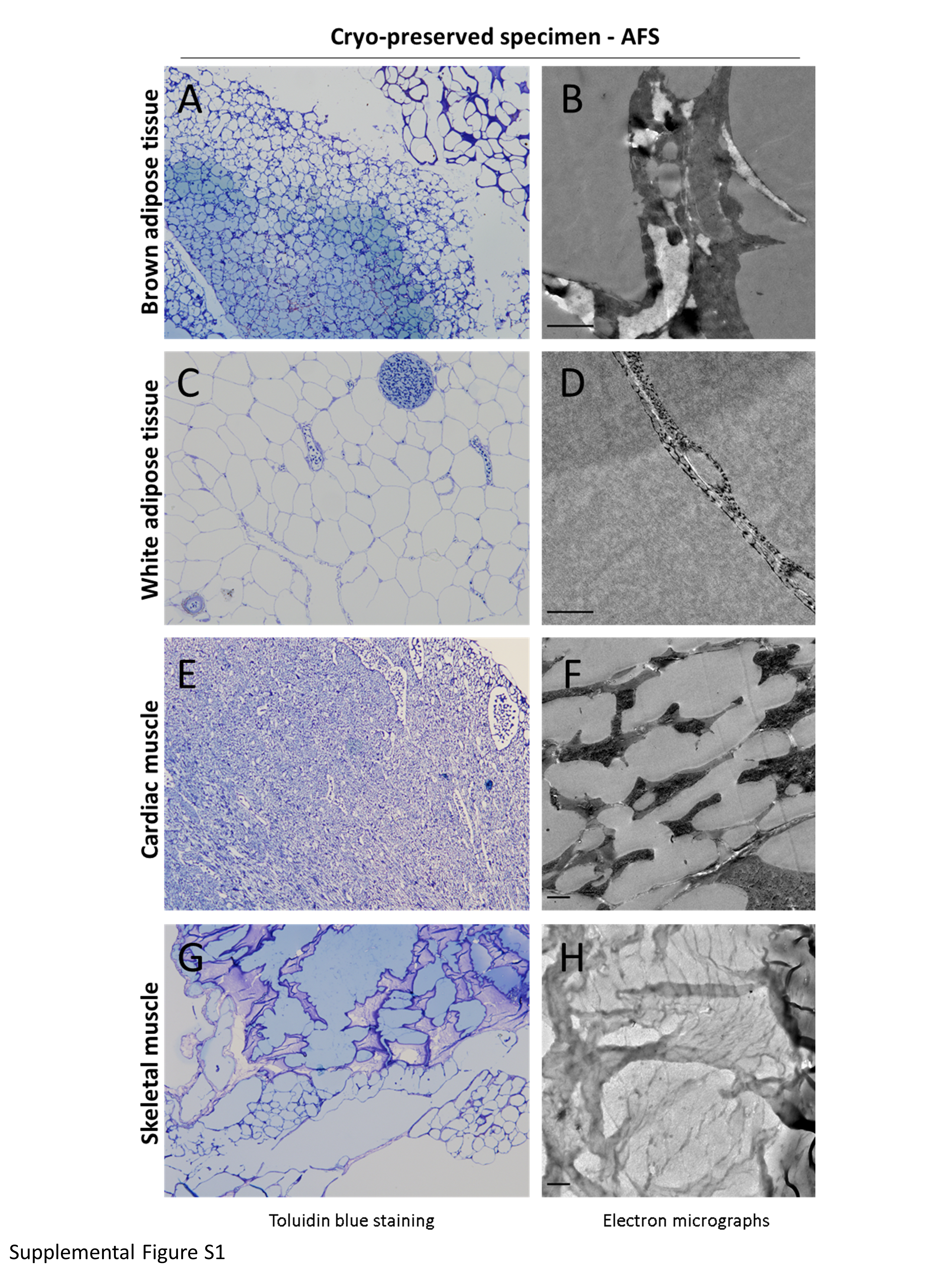

Supplement: Supplementary file 1 — Supplementary Figure S1 Toluidine blue staining and electron micrographs from cryo-preserved mouse samples after preparation with AFS. (A, B) brown adipose tissue. (C, D) white adipose tissue. (E, F) cardiac muscle. (G, H) skeletal muscle. Microscopic pictures show partly intact tissue structure with Toluidine blue staining (20X magnification), while electron micrographs show highly degenerated ultrastructure. Scale bar length 1µm (TIF 11955 KB) [file 418_2020_1952_MOESM1_ESM.tif]

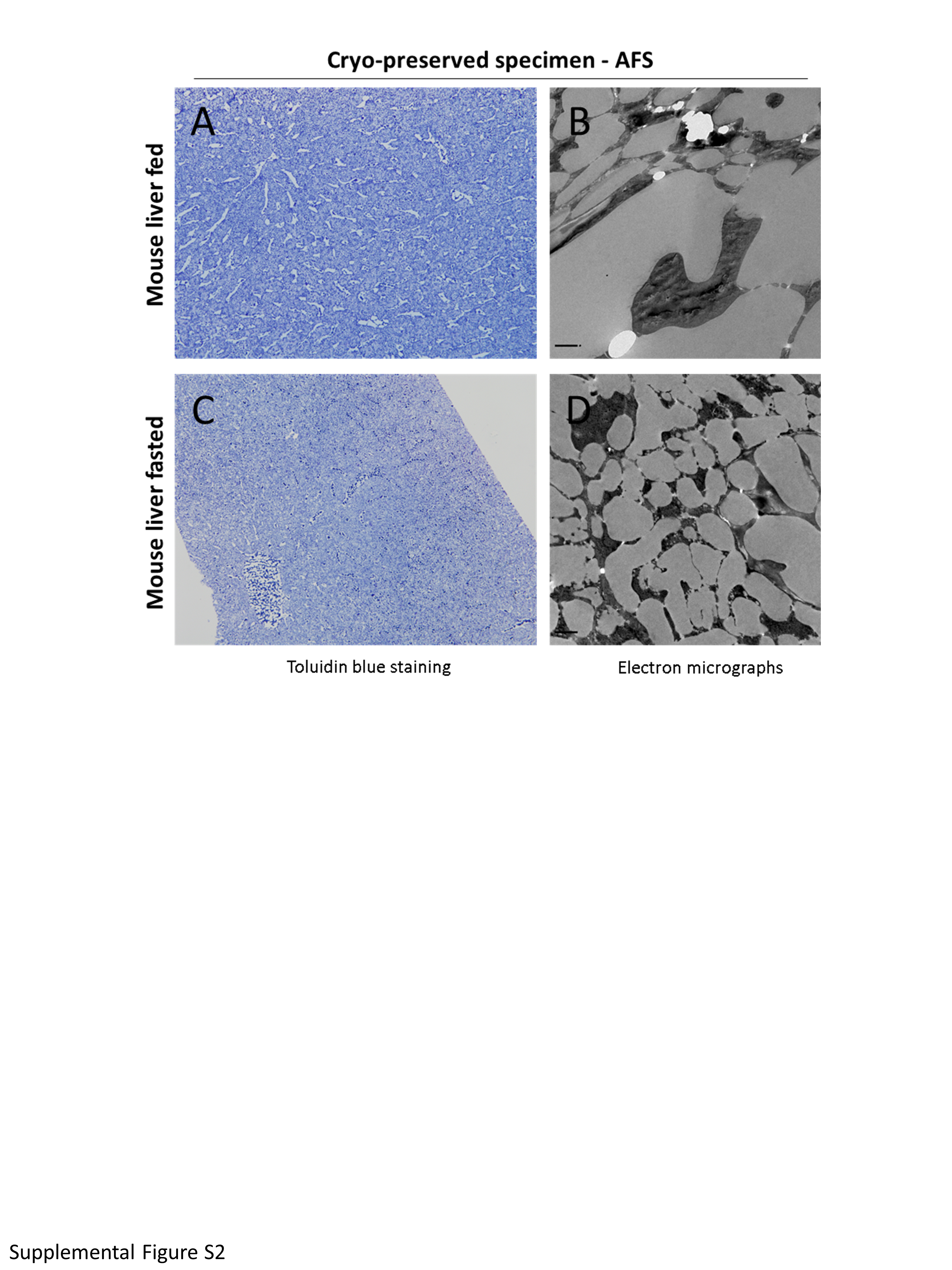

Supplement: Supplementary file 2 — Supplementary Figure S2 Electron micrographs from cryo-preserved mouse liver samples after preparation with AFS. (A, B) mouse liver fed. (C, D) mouse liver fasted. Toluidine blue staining 20X magnification. Scale bar length 1µm (TIF 7053 KB) [file 418_2020_1952_MOESM2_ESM.tif]

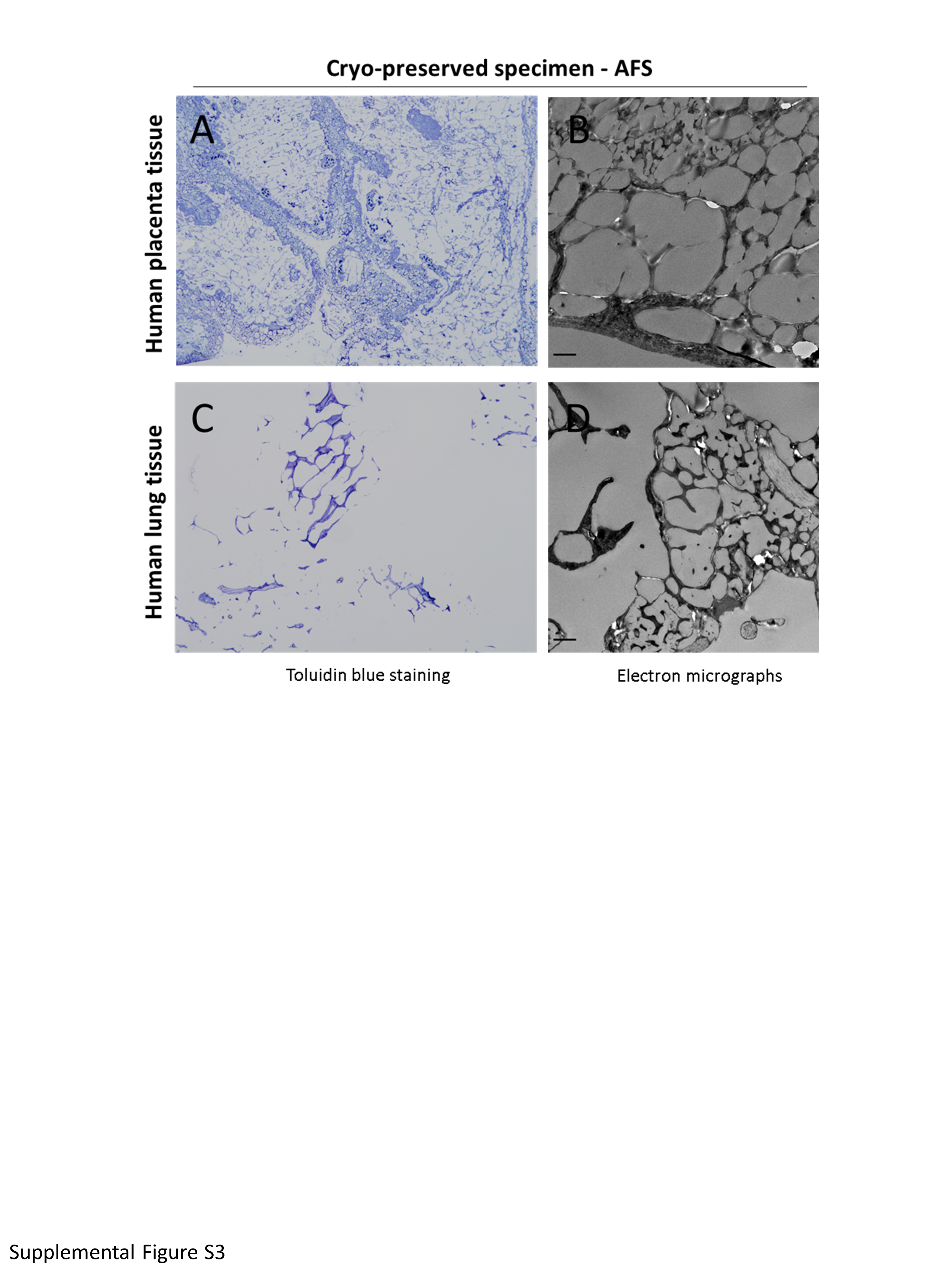

Supplement: Supplementary file 3 — Supplementary Figure S3 Electron micrographs from cryo-preserved human samples after preparation with AFS. (A, B) human placenta tissue. (C, D) human lung tissue. Toluidine blue staining 20X magnification. Scale bar length 1µm (TIF 5329 KB) [file 418_2020_1952_MOESM3_ESM.tif]
